# Supplementary material for: A high-resolution mRNA expression time course of embryonic development in zebrafish
Source: eLife. 2017 Nov 16;6:e30860. doi: 10.7554/eLife.30860 (PMC5690287; doi:10.7554/eLife.30860)
Supplement: Supplementary file 6. [file elife-30860-supp6.zip › biolayout-clusters-files/Cluster198-genes.html]

Cluster198


# Cluster198: Genes

| Ensembl ID | Gene Name | Chr | Start | End | Biotype |
| --- | --- | --- | --- | --- | --- |
| ENSDARG00000004177 | fam169ab | 21 | 15774775 | 15802712 | protein\_coding |
| ENSDARG00000005643 | gcat | 3 | 25859181 | 25867372 | protein\_coding |
| ENSDARG00000007398 | lrrk1 | 7 | 9281104 | 9471856 | protein\_coding |
| ENSDARG00000006332 | snx14 | 20 | 830757 | 873548 | protein\_coding |
| ENSDARG00000021455 | strbp | 10 | 9878451 | 10059882 | protein\_coding |
| ENSDARG00000045011 | tapbp.2 | 19 | 7119770 | 7151766 | protein\_coding |
| ENSDARG00000039356 | zgc:194209 | 4 | 4517335 | 4526276 | protein\_coding |
